# Geographical Analysis of Aneurysmal Subarachnoid Hemorrhage in Japan Utilizing Publically-Accessible DPC Database

**DOI:** 10.1371/journal.pone.0122467

**Published:** 2015-03-26

**Authors:** Toru Fukuhara

**Affiliations:** Department of Neurological Surgery, National Hospital Organization Okayama Medical Center, Okayama, Japan; Heinrich-Heine University, GERMANY

## Abstract

Since the launch of the novel medical reimbursement system Diagnosis Procedure Combination (DPC) in 2003 in Japan, inpatient data has been accumulated over time as part of a Japanese governmental nationwide database. This is partially accessible by the public, and this study examined the adequacy of this database as epidemiological research material by extracting the data relating to aneurysmal subarachnoid hemorrhage (aSAH) with special attention given to the limitations that this involves. Datasets after 2010 are considered suitable for analysis because of the numbers of participating hospitals and the analysis term. Extracting the data by prefecture, those with a continuously high aSAH incidence were Aomori, Iwate, Akita, Yamagata, Kochi and Kumamoto Prefectures, and those with low aSAH incidence were Kanagawa, Shiga, Kyoto, Shimane and Ehime Prefectures. Although these obtained results are informative, a publically-accessible DPC database has several limitations. Some limitations have been resolved: the analyzed term each year is now 12-months and the number of participating hospitals seems to have stabilized around 1700. However, other limitations such as masking the numbers in each hospital reporting less than 10 patients still exist, so careful and critical interpretation is necessary in utilizing a publically-accessible DPC database. Considering the potential of this database as material for epidemiological research, future analysis of the entire DPC database by qualified researchers is desirable.

## Introduction

Since April 2003, with 80 university hospitals and 2 national hospitals, the Japanese government has provided the Diagnosis Procedure Combination (DPC), a novel medical reimbursement system for hospitalization, in addition to the conventional fee-for-service payment [1–3]. The initial purpose of this system was to manage the inpatient costs by more uniform methods [1,2]; however, its promise as a nationwide database for epidemiological research has been indicated since the beginning of the DPC system [3]. This database has been partially accessible to the public on the homepage of the Japanese Ministry of Health, Labor and Welfare (JMHLW) [4], and it has the capacity to contribute to epidemiological analysis despite its fundamental limitations.

In this article, the general features and limitations of this publically-accessible database are addressed. To this end, the aneurysmal subarachnoid hemorrhage (aSAH) incidence was extracted from this database in each reported year. In order to grasp the geographical features of aSAH in Japan, the patient numbers were divided by 47 prefectures, and ranked to see whether there were any geographical tendencies. This article can form the basis of a preliminary report for future successive epidemiological analyses utilizing the DPC database in neurosurgical fields.

## Methods

This study requires no IRB approval, nor informed consent, since this was made only with a publically-accessible database through the Internet. The data available on that database is anonymized and de-identified prior to analysis. Several data files derived from the DPC database have been publically accessible since 2005 on the homepage of JMHLW [5]. On this page, several types of files, either in an Excel or PDF format, are displayed and the summarized annual patient numbers classified by the DPC code or Major Diagnostic Category (MDC) per each hospital are available for download. This database has three major limitations: 1) the number of participating hospitals submitting the DPC data has increased over time; 2) the analyzed term each year is not the same (for example, it started as a 4-month analysis in 2005, but it was a 12-month analysis in 2012); and 3) in the Excel file expressing the patient numbers of each hospital, patient numbers of less than 10 in the segmentalized fields according to the DPC code are masked. Due to these three limitations, analysis with the publically-accessible DPC database will not show the actual figures of aSAH in Japan; hence, an annual comparison will not confirm whether the figures are increasing. However, aSAH tend to be concentrated in large institutes participating in the DPC system, and comparison between prefectures in the same year will reveal meaningful insights regarding the geographical differences. To address this issue, the data of the prefecture in which each hospital is located was added to the downloaded Excel files, and then the number of aSAH was calculated according to each prefecture.

The annual DPC database report has been uploaded for the following years on the homepage of JMHLW. The details and URL of the files used for data extraction are shown in S1–S4 Appendix. In order to grasp the general features of the DPC database, the number of participating hospitals and the analyzed term were extracted per each year (S1 Appendix). The total patient number composing each annual DPC database can be calculated from the patient numbers of hospitals grouped according to the year of participating in the DPC system (S2 Appendix). The total patient number of aSAH in the annual DPC database can be obtained with the number in several DPC codes indicating aSAH, and the files used for these calculations are shown in S3 Appendix with the corresponding DPC codes.

In order to address the patient numbers of aSAH by prefecture, Excel files expressing the patient numbers in each participating hospital were utilized, as shown in S4 Appendix. In these files, a number of patients less than 10 is not reported, and the numbers obtained by summing up the patients of reporting hospitals result in smaller numbers than the total obtained in the above with files in S3 Appendix. The percentage of unreported numbers in Excel files reporting the patient numbers of each hospital was obtained by (total number—summed-up number) X100/ (total number), and named the “masked rate.” It is important to recognize this “masked rate” between the total patient number and summed-up patient number of hospitals reporting 10 or more patients in order to grasp the impact on the analysis. Then the population of each prefecture was extracted from the homepage of the Statistics Bureau, Ministry of Internal Affairs and Communications [6]. With these data, the number of aSAH per 100,000 population can be obtained in each prefecture. The aSAH incidence was ranked by prefecture in each year.

## Results

Table 1 shows the results of the data extraction. In the first two years, 2005 and 2006, the number of participating hospitals was less than 1,000, and the total number of reported aSAH was less than 5,000, so these data are considered inadequate for further analysis. Between 2007 and 2009, there were still several inadequate aspects for the annual evaluation; the 6-month term and the “masked rate” of aSAH, larger than 50%, although the number of participating hospitals increased to around 1500 and the total number of reported aSAH was close to 10,000. As for 2010, the “masked rate” is less than 40%, therefore, the data of 2010 can be utilized for the analysis even though the term is 9 months. In 2011 and 2012, the analyzed term is 12 months and the “masked rate” is less than 35%. Considering these features, for the analysis of prefectural differences, the data between 2010 and 2012 were used.

**Table 1:** Numbers of aSAH in publically-accessible DPC database.

Year (Report date)	No. of Hps	Duration (term)	No. of Patients	Total aSAH	Summed-up aSAH* (No. of Hps)	Masked Rate
2005 (4/27/06)	372	4 months (Jul-Oct)	987,726	2,192	824 (52)	62.4%
2006 (6/22/07)	731	6 months (Jul-Dec)	2,578,442	4,622	2,988 (162)	35.4%
2007 (5/9/08)	1,428	6 months (Jul-Dec)	3,944,700	9,093	4,083 (230)	55.1%
2008 (5/14/09)	1,559	6 months (Jul-Dec)	4,229,184	9,375	4,342 (246)	53.7%
2009 (6/30/10)	1,607	6 months (Jul-Dec)	4,379,114	9,673	4,429 (251)	54.2%
2010 (11/7/11)	1,648	9 months (Jul-Mar)	6,769,203	14,660	8,868 (397)	39.5%
2011 (8/21/12)	1,634	12 months (Apr-Mar)	8,780,880	17,339	11,605 (463)	33.1%
2012 (9/20/13)	1,774	12 months (Apr-Mar)	9,249,923	18,033	12,282 (482)	31.9%

*Numbers are calculated by summing up the patient numbers from hospitals reporting 10 or more patients, the numbers of which are indicated in parentheses.

Hps = hospitals.

Table 2 indicates the top 10 prefectures and prefectures within the 10th place from the bottom in terms of aSAH incidence among hospitals reporting 10 or more patients in each year, which revealed a large difference between prefectures; from 4.1 in Toyama to 21.6 in Aomori in 2012. Six prefectures were continuously ranked within the top 10 for 3 years: Aomori (ranked first for 3 years), Iwate (ranked 2-3-2), Akita (ranked fourth for 3 years), Yamagata (ranked 8-2-6), Kochi (ranked 5-5-8) and Kumamoto (ranked 3-6-3). In contrast, 5 prefectures have less aSAH continuously for 3 years within the 10th place from the bottom: Kanagawa (ranked 39-41-41), Shiga (ranked 45-43-46), Kyoto (ranked 46-44-38), Shimane (ranked 42-47-43) and Ehime (ranked 43-46-44). For these prefectures, there seems to be a certain tendency in terms of aSAH incidence. The details of obtained aSAH incidences of all 47 prefectures for these 3 years are shown in S5 Appendix. As for the nationwide aSAH incidence, in 2012, the total population in Japan was 127,515,000 and the reported number of aSAH patients was 18,033, thus, the incidence per 100,000 people was 14.1 from the entire DPC database (no effect of “masked rate”).

**Table 2:** Prefectural rank of aSAH incidence calculated with hospitals reporting 10 or more patients.

	Prefecture (Incidence of aSAH per 100,000 people)		
Rank	2010*	2011	2012
1	Aomori (24.4)	Aomori (18.6)	Aomori (21.6)
2	Iwate (16.3)	Yamagata (18.5)	Iwate (17.1)
3	Kumamoto (15.0)	Iwate (15.8)	Kumamoto (15.3)
4	Akita (13.1)	Akita (14.2)	Akita (14.2)
5	Kochi (12.9)	Kochi (13.6)	Gunma (13.8)
6	Tottori (12.5)	Kumamoto (12.8)	Yamagata (13.5)
7	Tochigi (12.0)	Tokushima (12.2)	Yamaguchi (13.4)
8	Yamagata (11.9)	Wakayama (11.9)	Kochi (13.0)
9	Okayama (11.6)	Nagano (11.9)	Fukuoka (12.2)
10	Wakayama (11.6)	Nagasaki (10.8)	Tochigi (11.5)
38	Yamanashi (7.0)	Nara (7.0)	Kyoto (7.7)
39	Kanagawa (6.5)	Osaka (6.8)	Kagoshima (7.7)
40	Toyama (6.1)	Fukui (6.7)	Osaka (7.4)
41	Miyazaki (6.0)	Kanagawa (6.7)	Kanagawa (7.3)
42	Shimane (6.0)	Yamanashi (5.8)	Ishikawa (7.2)
43	Ehime (5.7)	Shiga (5.8)	Shimane (6.9)
44	Ishikawa (5.4)	Kyoto (5.2)	Ehime (6.5)
45	Shiga (4.8)	Oita (5.0)	Nara (6.5)
46	Kyoto (4.3)	Ehime (4.9)	Shiga (4.3)
47	Oita (3.1)	Shimane (4.4)	Toyama (4.1)

*The number is estimated by multiplying 4/3 times, since the analyzed term is 9 months.

## Discussion

Analyzing large public health data sets for certain medical issues can uncover the effects of exposure that may have small effects on individuals but large cumulative effects on populations [7]. With the rapid expansion of electronic medical record usage, much larger amounts of medical information can be prepared for medical analysis; however, large data is often highly complex because of its high volume and its various sources [8]. The data on the DPC system, although the system is huge, are relatively free from these biases, with the uniform reporting format for coding [9] and considerably fewer coding errors due to its nature relating to the hospital fee charge. The significance of facilitating such large databases is well recognized, however; to the best of my knowledge, no national database in the world with sufficient medical information on uniform reporting formats covers the entire population. In the US, the largest inpatient care database is the Nationwide Inpatient Sample (NIS) with approximately 8 million admissions each year [10], which covers one-fifth of all inpatient admissions to non-federal US hospitals [11,12]. As for the DPC database, which contains more than 9 million admissions in 2012, the number of in-patient beds under DPC systems was approximately half a million as of 2012, among approximately 0.9 million in total in Japan [13]. This means approximately half of all inpatient beds in Japan were covered by the hospitals participating in this system, moreover it has been reported that approximately 90% of the total acute inpatient hospitalization is covered by the DPC system [4,14]. The DPC database has promising potential as material for epidemiological analysis from a worldwide perspective.

Although analysis of the DPC database can be informative, there are several significant limitations to this database as shown in Table 3. One of the limitations specific to the publically-accessible DPC database, the various analyzed terms (No.1), will disappear in the future, since it has been 12 months analysis for these two years and probably will be the same in the future. Another significant limitation specific to publically-accessible data is the “masked rate,” that is, patient numbers less than 10 recorded in each hospital are masked (No.2) and the reason for masking is probably for the hospital to avoid the hospital ranking by patient volume alone, irrespective of the outcome [9]. So when analyzing data consisting of each hospital patient number, there are unreported patients, and the unreported rate should be recognized as a bias of the study. These unreported numbers of patients may not be uniformly distributed among all prefectures (indicated as limitation No.9); however, excessive unproportional deviations of these masked patients in particular prefectures are not realistic, and if the “masked rate” is considerably small, the analysis will be recognized as meaningful. Among the limitations specific to the DPC database itself (namely the entire database including the data not open to the public), the increasing number of participating hospitals (No.3) seems to be somewhat stable after 2007. Unreported aSAH patients because of being treated at the hospital out of the DPC system (No. 4) do exist; however, the number should not be significantly large since the admission due to aSAH is mostly on an emergency basis, and up to 90% of emergency admissions are considered to be managed at DPC participating hospitals [4,14]. Other limitations, listed as No. 5 (double counts), No.6 (coding inconsistency) and No. 7 (misdiagnosis), will occur in any analyses with a huge database and the larger the data volume, the lower the impact of these biases will become. Some patients do visit hospitals in different prefectures (No.8); however, the number is probably negligible, since the majority of patients with aSAH are taken to hospitals by ambulance in the same prefectures in which the patients live.

**Table 3:** Considerable limitations in current study.

Limitation specific to publically-accessible DPC database
1. The analyzed term is not annual during the initial 6 years.
2. Patient numbers less than 10 are masked.
Limitation specific to DPC database itself
3. The number of participating hospitals is increasing.
4. Some unreported patients are treated by hospitals not in the DPC database.
5. Double counts of the same patient occurr in different hospitals.
6. The manner of patient coding is partially inconsistent with several revisions.
7. Some aSAH are unreported due to misdiagnosis.
Limitation inherent to methodology
8. Some patients are treated in a different prefecture.
9. Data from different prefectures is biased to different extents by the above-mentioned limitations.

Physicians in Japan are aware that many items regarding the patient’s condition on every admission are sent to the government to make up the DPC database, including the presence of comorbid diseases, the consciousness level at admission and the detailed outcome, although most of this information is non-public. There are several articles utilizing these detailed non-public DPC datasets collected from only specific hospitals joining a certain research group [4,14–16], but not from the entire database the government protects. Studies utilizing only the publically-accessible DPC database, as conducted in this article, are scarce [17], probably due to the difficulty in analyzing the data with the “masked rate”; however, the great assets of the publically-accessible database are that it is nationwide and free from the ethical issues, since personal identifiable information is protected, and approved by the government. With these free-access but restricted data, numerous medical analyses are possible, some of which are expected to suggest facts related to public health of worldwide significance. Researchers proposing medically significant hypotheses obtained with these publically-accessible data should be allowed to access the entire body of this database (which contains much more detailed medical information) for their further researches. When utilizing a large database with possible identifiable information of the patients, some access restrictions are inevitable [7], so the establishment of appropriate guidelines on qualifications for accessing this database is desired.

Even with the above-mentioned biases, the current study reveals epidemiologically interesting facts about the regional differences of aSAH incidence. The incidence of aSAH is not uniform across the country; it is the highest in Aomori. There is expected to be quite a large deviation in aSAH incidence even in the same country, and such a large difference cannot be explained solely by the biases inherent in using the DPC database. Among the six highly-ranked prefectures continuously for 3 years with aSAH incidence, four prefectures (Aomori, Iwate, Akita and Yamagata) are in the northern territory, and two (Kochi and Kumamoto) in the south. Although climactic factors are expected to affect aneurysmal rupture [18,19], the climate does not completely explain the regional differences, since in several other northern territories, such as Hokkaido or Miyagi, aSAH is not so frequent, and the climate in Kochi or Kumamoto, where high aSAH incidence is observed, is very different from that in the northern territory. Other possible factors resulting in the regional difference are genetic factors [20], dietary habits in the region [21], or other unknown environmental factors.

aSAH incidence in Japan is believed to be higher than that in other countries in the world, reported as 22.7 per 100,000 people by de Rooij et al. [22], which is far higher than the number obtained in this article; 14.1 in 2012 from the entire DPC database. The number reported by de Rooij et al. may not be accurate since the number was calculated not from the entire area of Japan, but from former reports from certain regions in Japan such as Izumo city [23], Shimokita [24] or Kumamoto [25], and the reported year of those studies was around the mid-1990s. Although the aSAH incidence obtained in this study may be lower than actual incidence due to the fact that the DPC system only covers approximately half of the inpatient beds in Japan, the DPC system is believed to cover approximately 90% of the total acute inpatient hospitalization [4,14], thus the number in this study will be close to the actual figure. However the calculated incidence cannot be the actual figure, as there are considerable biases in obtaining aSAH incidence from a database, including sampling errors and regional time trends, and the exact incidence of aSAH can be obtained only with a population-based study.

## Conclusion

The introductory study with a publically-accessible DPC database was presented by examining aSAH incidence in Japan. The results were suggestive of large regional variations in aSAH incidence. Although several significant limitations of the DPC database have been revealed, its potential as material for epidemiological analysis warrants further study.

## Supporting Information

S1 Appendix PDF files describing the analyzed term and total number of participating hospitals. (DOCX)

S2 Appendix Files describing total patient number composing each annual DPC database. (DOCX)

S3 Appendix Files for obtaining the total patient number of aSAH in the annual DPC database. (DOCX)

S4 Appendix Excel files used to address the patient numbers of aSAH by prefecture. (DOCX)

S5 Appendix Prefectural aSAH incidence calculated with hospitals reporting 10 or more patients. (DOCX)
