# Supplementary material for: Geographical Analysis of Aneurysmal Subarachnoid Hemorrhage in Japan Utilizing Publically-Accessible DPC Database
Source: PLoS One. 2015 Mar 26;10(3):e0122467. doi: 10.1371/journal.pone.0122467 (PMC4374883; doi:10.1371/journal.pone.0122467)
Supplement: S1 Appendix — (DOCX) [file pone.0122467.s001.docx]

**Appendix S1. PDF files describing the analyzed term and total number of participating hospitals***

2005 document D-2 / 1,435KB

http://www.mhlw.go.jp/shingi/2006/04/dl/s0427-3b.pdf

2006 document D-1 / 2,558KB

http://www.mhlw.go.jp/shingi/2007/06/dl/s0622-7b.pdf

2007 document D-1 / 226KB

http://www.mhlw.go.jp/shingi/2008/05/dl/s0509-3a.pdf

2008 document D-1 / 261KB

http://www.mhlw.go.jp/shingi/2009/05/dl/s0514-6a.pdf

2009 document D-1 / 347KB

http://www.mhlw.go.jp/shingi/2010/06/dl/s0360-7a.pdf

2010 reference material 2-(1) / 140KB

http://www.mhlw.go.jp/stf/shingi/2r9852000001u23a-att/2r9852000001u94y.pdf

2011 reference material 2-(1) / 235KB

http://www.mhlw.go.jp/stf/shingi/2r9852000002hs9l-att/2r9852000002hslq.pdf

2012 reference material 2-(1) / 235KB

http://www.mhlw.go.jp/file/05-Shingikai-12404000-Hokenkyoku-Iryouka/0000023553.pdf

*The numbers are described in the first page every year.
