# Supplementary material for: Geographical Analysis of Aneurysmal Subarachnoid Hemorrhage in Japan Utilizing Publically-Accessible DPC Database
Source: PLoS One. 2015 Mar 26;10(3):e0122467. doi: 10.1371/journal.pone.0122467 (PMC4374883; doi:10.1371/journal.pone.0122467)
Supplement: S2 Appendix — (DOCX) [file pone.0122467.s002.docx]

**Appendix S2. Files describing total patient number composing each annual DPC database***

2005 document D-2 / 1,435KB PDF file (page 32)

http://www.mhlw.go.jp/shingi/2006/04/dl/s0427-3b.pdf

2006 document D-1 / 180KB Excel file

http://www.mhlw.go.jp/shingi/2007/06/xls/s0622-7j.xls

2007 reference material 1 / 3,856KB Excel file

http://www.mhlw.go.jp/shingi/2008/05/xls/s0509-3f.xls

2008 reference material 1 / 4,737KB Excel file

http://www.mhlw.go.jp/shingi/2009/05/xls/s0514-6f.xls

2009 document 2 / 590KB Excel file

http://www.mhlw.go.jp/shingi/2010/06/xls/s0360-7a.xls

2010 reference material 1-(3) / 843KB Excel file

http://www.mhlw.go.jp/stf/shingi/2r9852000001u23a-att/2r9852000001u911.xls

2011 reference material 1-(3) / 856KB Excel file

http://www.mhlw.go.jp/stf/shingi/2r9852000002hs9l-att/2r9852000002hsir.xls

2012 reference material 1-(3) / 1119KB Excel file

http://www.mhlw.go.jp/file/05-Shingikai-12404000-Hokenkyoku-Iryouka/0000023537.xls

*The numbers are calculated by summing up the patient numbers of hospitals grouped according to the year of participating in the DPC system.
