# Supplementary material for: Geographical Analysis of Aneurysmal Subarachnoid Hemorrhage in Japan Utilizing Publically-Accessible DPC Database
Source: PLoS One. 2015 Mar 26;10(3):e0122467. doi: 10.1371/journal.pone.0122467 (PMC4374883; doi:10.1371/journal.pone.0122467)
Supplement: S3 Appendix — (DOCX) [file pone.0122467.s003.docx]

**Appendix S3. Files for obtaining the total patient number of aSAH in the annual DPC database***

2005 document D-4-(8) / 47,941KB PDF file

<http://www.mhlw.go.jp/shingi/2006/04/dl/s0427-3d106.pdf>

DPC codes of aSAH (page 18-25)

0100201xxxxxxx, 0100203099x0xx, 0100203099x1xx, 0100203002xxxx, 0100203001xxxx, 0100203199x1xx, 0100203102x1xx, 0100203101xxxx

2006 document D-1, supplement 2 / 54,241KB PDF file

<http://www.mhlw.go.jp/shingi/2007/06/dl/s0622-7d.pdf>

DPC codes of aSAH (page 18-28)

010020x099x1xx, 010020x003x0xx, 010020x003x1xx, 010020x002x1xx, 010020x001x0xx, 010020x001x1xx, 010020x199x1xx, 010020x103x1xx, 010020x102x1xx, 010020x101x10x, 010020x101x11x

2007 reference material 1 / 1,304KB Excel file

<http://www.mhlw.go.jp/shingi/2008/05/xls/s0509-3e.xls>

2008 reference material 1 / 1,577KB Excel file

<http://www.mhlw.go.jp/shingi/2009/05/xls/s0514-6e.xls>

2009 reference material 1 / 2,014KB Excel file

<http://www.mhlw.go.jp/shingi/2010/06/xls/s0360-7g.xls>

2010 reference material 1-(13) / 2,982KB

<http://www.mhlw.go.jp/stf/shingi/2r9852000001u23a-att/2r9852000001u93n.xls>

2011 reference material 1-(13) / 6,626KB

<http://www.mhlw.go.jp/stf/shingi/2r9852000002hs9l-att/2r9852000002hskp.xls>

2012 reference material 1-(13) / 5,791KB

<http://www.mhlw.go.jp/file/05-Shingikai-12404000-Hokenkyoku-Iryouka/0000023547.xls>

DPC codes of aSAH (2007 – 2012)

010020x099x0xx, 010020x099x1xx, 010020x097x0xx, 010020x097x1xx, 010020x003x0xx, 010020x003x1xx, 010020x002x0xx, 010020x002x1xx, 010020x001x0xx, 010020x001x1xx, 010020x199x0xx, 010020x199x1xx, 010020x197x0xx, 010020x197x1xx, 010020x103x0xx,

010020x103x1xx, 010020x102x0xx, 010020x102x1xx, 010020x101x0xx, 010020x101x1xx

*The total patient numbers are obtained by summing up the numbers in the corresponding DPC codes.
