# Supplementary material for: Geographical Analysis of Aneurysmal Subarachnoid Hemorrhage in Japan Utilizing Publically-Accessible DPC Database
Source: PLoS One. 2015 Mar 26;10(3):e0122467. doi: 10.1371/journal.pone.0122467 (PMC4374883; doi:10.1371/journal.pone.0122467)
Supplement: S4 Appendix — (DOCX) [file pone.0122467.s004.docx]

**Appendix S4. Excel Files used to address the patient numbers of aSAH by prefecture**

2005 document D-4-(2) / 2,486KB

http://www.mhlw.go.jp/shingi/2006/04/xls/s0427-3d31.xls

2006 document D-1 / 6,271KB

http://www.mhlw.go.jp/shingi/2007/06/xls/s0622-7m.xls

2007 reference material 2-(8)-01 / 4,724KB

http://www.mhlw.go.jp/shingi/2008/05/xls/s0509-3z.xls

2008 reference material 2-(8)-01 / 5,146KB

http://www.mhlw.go.jp/shingi/2009/05/xls/s0514-6ag.xls

2009 reference material 2-01 / 5,320KB

http://www.mhlw.go.jp/shingi/2010/06/xls/s0360-7n_01.xls

2010 reference material 2-(9) -MDC01 / size not shown

http://www.mhlw.go.jp/stf/shingi/2r9852000001ue1v-att/2r9852000001ue4m.xls

2011 reference material 2-(9)-MDC01 / 5,548KB

http://www.mhlw.go.jp/stf/shingi/2r9852000002hs9l-att/2r9852000002hsv9.xls

2012 reference material 2-(9)-MDC01 / 6,115KB

http://www.mhlw.go.jp/file/05-Shingikai-12404000-Hokenkyoku-Iryouka/0000023588.xls
