# Supplementary material for: Geographical Analysis of Aneurysmal Subarachnoid Hemorrhage in Japan Utilizing Publically-Accessible DPC Database
Source: PLoS One. 2015 Mar 26;10(3):e0122467. doi: 10.1371/journal.pone.0122467 (PMC4374883; doi:10.1371/journal.pone.0122467)
Supplement: S5 Appendix — (DOCX) [file pone.0122467.s005.docx]

Appendix S5: Prefectural aSAH incidence calculated with hospitals reporting 10 or more patients

Prefecture Incidence of aSAH per 100,000 people (Number/Population^*^/Hp)

2010^**^ *rank* 2011 *rank* 2012 *rank*

Hokkaido 9.6 (398/5,506/20) *20* 10.4 (568/5,486/25) *15* 8.4 (458/5,460/17) *35*

Aomori 24.4 (251/1,373/7) *1* 18.6 (253/1,363/6) *1* 21.6 (292/1,350/7) *1*

Iwate 16.3 (163/1,330/7) *2* 15.8 (207/1,314/7) *3* 17.1 (223/1,303/6) *2*

Miyagi 9.7 (171/2,348/5) *19* 9.4 (219/2,327/5) *24* 9.7 (225/2,325/5) *25*

Akita 13.1 (107/1,086/6) *4* 14.2 (153/1,075/8) *4* 14.2 (151/1,063/7) *4*

Yamagata 11.9 (104/1,169/4) *8* 18.5 (215/1,161/8) *2* 13.5 (156/1,152/8) *6*

Fukushima 11.0 (168/2,029/5) *12* 10.4 (206/1,990/8) *16* 10.4 (203/1,962/7) *16*

Ibaraki 7.2 (160/2,970/7) *36* 7.0 (208/2,958/8) *37* 9.7 (285/2,943/10) *24*

Tochigi 12.0 (180/2,008/6) *7* 10.4 (208/2,000/6) *14* 11.5 (228/1,992/6) *10*

Gunma 8.9 (134/2,008/8) *24* 10.5 (211/2,001/8) *11* 13.8 (274/1,992/12) *5*

Saitama 9.2 (495/7,195/19) *21* 9.0 (648/7,207/20) *29* 9.0 (648/7,212/21) *30*

Chiba 8.3 (388/6,216/15) *31* 9.2 (573/6,214/19) *27* 10.3 (638/6,195/20) *19*

Tokyo 11.0 (1088/13,159/40) *14* 9.6 (1266/13,196/43) *21* 10.0 (1316/13,230/50) *22*

Kanagawa 6.5 (442/9,048/23) *39* 6.7 (608/9,058/27) *41* 7.3 (658/9,067/29) *41*

Niigata 8.7 (154/2,374/7) *26* 10.2 (241/2,362/11) *17* 8.7 (204/2,347/8) *33*

Toyama 6.1 (50/1,093/2) *40* 9.3 (101/1,088/4) *26* 4.1 (44/1,082/2) *47*

Ishikawa 5.4 (47/1,170/3) *44* 7.7 (90/1,166/6) *33* 7.2 (84/1,163/4) *42*

Fukui 7.1 (43/806/3) *37* 6.7 (54/803/3) *40* 9.9 (79/799/3) *23*

Yamanashi 7.0 (45/863/2) *38* 5.8 (50/857/2) *42* 8.2 (70/852/2) *36*

Nagano 10.2 (165/2,152/9) *18* 11.9 (235/2,142/11) *9* 10.6 (225/2,132/11) *12*

Gifu 8.3 (130/2,081/7) *30* 7.3 (152/2,071/8) *36* 10.0 (206/2,061/9) *20*

Shizuoka 8.9 (252/3,765/14) *23* 9.6 (358/3,749/17) *23* 9.2 (344/3,735/16) *27*

Aichi 10.7 (593/7,411/24) *15* 10.1 (747/7,416/27) *18* 10.4 (774/7,427/28) *15*

Mie 8.3 (115/1,855/7) *32* 10.5 (194/1,847/9) *13* 9.0 (166/1,840/9) *28*

Shiga 4.8 (51/1,411/3) *45* 5.8 (82/1,414/5) *43* 4.3 (61/1,415/4) *46*

Kyoto 4.3 (84/2,636/5) *46* 5.2 (138/2,632/7) *44* 7.7 (202/2,625/7) *38*

Osaka 7.3 (484/8,865/22) *34* 6.8 (598/8,861/27) *39* 7.4 (656/8,856/30) *40*

Hyogo 8.6 (359/5,588/17) *28* 7.6 (423/5,582/19) *34* 8.8 (488/5,571/20) *31*

Nara 8.9 (93/1,401/4) *25* 7.0 (97/1,396/5) *38* 6.5 (90/1,390/5) *45*

Wakayama 11.6 (87/1,002/4) *10* 11.9 (118/995/4) *8* 11.4 (113/988/5) *11*

Tottori 12.5 (55/589/4) *6* 8.2 (48/585/4) *30* 10.3 (60/582/4) *17*

Shimane 6.0 (32/717/2) *42* 4.4 (31/712/1) *47* 6.9 (49/707/2) *43*

Okayama 11.6 (169/1,945/8) *9* 9.4 (182/1,941/7) *25* 10.5 (204/1,936/7) *13*

Hiroshima 8.4 (180/2,861/8) *29* 8.0 (228/2,855/10) *31* 10.0 (284/2,848/13) *21*

Yamaguchi 11.0 (120/1,451/6) *13* 9.2 (132/1,442/6) *28* 13.4 (192/1,431/8) *7*

Tokushima 7.3 (43/785/2) *33* 12.2 (95/780/3) *7* 8.5 (66/776/3) *34*

Kagawa 10.4 (78/996/4) *17* 7.4 (73/992/4) *35* 9.0 (89/989/4) *29*

Ehime 5.7 (61/1,431/2) *43* 4.9 (70/1,423/2) *46* 6.5 (92/1,415/2) *44*

Kochi 12.9 (74/764/4) *5* 13.6 (103/758/4) *5* 13.0 (98/752/4) *8*

Fukuoka 10.5 (398/5,072/22) *16* 10.0 (509/5,079/21) *19* 12.2 (620/5,085/26) *9*

Saga 8.6 (55/850/4) *27* 9.6 (81/847/5) *22* 7.7 (65/843/4) *37*

Nagasaki 9.1 (97/1,427/5) *22* 10.8 (153/1,417/7) *10* 10.4 (147/1,408/8) *14*

Kumamoto 15.0 (205/1,817/2) *3* 12.8 (232/1,813/7) *6* 15.3 (276/1,807/7) *3*

Oita 3.1 (28/1,197/1) *47* 5.0 (59/1,191/3) *45* 8.7 (103/1,185/5) *32*

Miyazaki 6.0 (51/1,135/1) *41* 10.5 (119/1,131/4) *12* 10.3 (116/1,126/5) *18*

Kagoshima 11.3 (145/1,706/6) *11* 7.8 (133/1,699/4) *32* 7.7 (130/1,690/4) *39*

Okinawa 7.3 (76/1,393/6) *35* 9.7 (136/1,401/8) *20* 9.2 (130/1,409/8) *26*

*The population is expressed in thousands.

**The number is estimated by multiplying 4/3 times, since the analyzed term is 9 months.

Hp = hospital.
